# Comprehensive characterization of malignant phyllodes tumor by whole genomic and proteomic analysis: biological implications for targeted therapy opportunities

**DOI:** 10.1186/1750-1172-8-112

**Published:** 2013-07-30

**Authors:** Denis L Fontes Jardim, Anthony Conley, Vivek Subbiah

**Affiliations:** 1 Department of Investigational Cancer Therapeutics, The University of Texas MD Anderson Cancer Center, 1515 Holcombe Blvd. FC8.3038, Box 0455, Houston, TX 77030, USA; 2 Department of Sarcoma Medical Oncology, Division of Cancer Medicine, The University of Texas MD Anderson Cancer Center, 1515 Holcombe Blvd., Houston, TX 77030, USA

**Keywords:** Phyllodes tumor, Phyllodes sarcoma, Sarcoma, Targeted therapy, Chemotherapy, SPARC, TLE3, NRAS, P53, P13K, Mutation, Rare disease, Breast cancer, Breast sarcoma, Next generation sequencing, CLIA, Immunohistochemistry, Morphoproteomics, Proteomics, Whole genome sequencing

## Abstract

**Background:**

Phyllodes tumors are uncommon breast tumors that account for less than 0.5% of all breast malignancies. After metastases develop, the prognosis is poor, with very few patients living more than 1 year. The biology of this unusual cancer is not understood and, consequently, no potential targets for treatments are currently available. There has been an exponential increase in the number of commercially available tumor profiling services. Herein, we report a case of metastatic malignant phyllodes tumor for which a comprehensive molecular analysis was performed by using Clinical Laboratory Improvement Amendments (CLIA)-certified labs, providing new insights into the potential opportunities for molecularly targeted therapies for this extremely rare disease.

**Methods:**

Next-generation sequencing was performed by using the FoundationOne™ platform (Foundation Medicine, Cambridge, MA). Whole-genome array-based comparative genomic hybridization (array CGH) was performed by using the DNAarray™ (CombiMatrix Diagnostics, Irvine, CA). Immunohistochemical and morphoproteomics analysis were performed at Consultative proteomics^®^, The University of Texas, UT Health Medical School, Houston,TX (Robert E Brown Lab); Clarient Diagnostics, Aliso Viejo, CA; and Caris Life Sciences Target one, Irving, TX, USA.

**Results:**

Next-generation sequencing showed 3 aberrant genes: activating mutation Q61L on *NRAS*; inactivating mutations Q504* and K740* on *RB1*; and *TP53* loss. Whole-genome array-based comparative genomic hybridization (array CGH) revealed amplifications of chromosome (chr.) 1 (*CKS1B* gene), chr. 8 (*MYC* gene), and chr. 9 (*CDKN2A* gene) Deletions of chr. 17 (*TP53*), chr. 10 (*GATA3*), chr. 11 (*FGF4* and *CCND1* genes), and chr.22 (*PDGFβ*). Immunohistochemical analysis for relevant markers showed a positive staining for transducing-like enhancer of split (TLE) 3; secreted protein acidic and rich in cysteine (SPARC) was expressed at 2-3+ in the cytoplasm of the tumors cells, whereas mammalian target of rapamycin (mTOR) was expressed up to 2+ in the nuclei of the tumor cells.

**Conclusions:**

We describe for the first time an *NRAS* mutation with concomitant activation of *PI3K*/*Akt*/*mTOR* in phyllodes tumor. We also found markers for sensitivity to taxane-based therapies, especially albumin-bound paclitaxel. Exploring the biology of rare malignancies by CLIA certified labs may be reasonable strategy for the development of targeted treatments.

## Background

Phyllodes tumors are uncommon breast tumors that account for less than 0.5% of all breast malignancies [1]. Histologically, a phyllodes tumor is a fibroepithelial neoplasia subdivided into benign, borderline, and malignant subtypes. Malignant phyllodes are the most aggressive subtype in this spectrum and correspond to about 20% of all phyllodes tumors [2]. They are characterized by high mitotic rates, marked stromal cellularity and atypia, and infiltrative margins [3]. In contrast to benign and borderline lesions, malignant tumors can metastasize in up to 22% of cases [1,4-6]. After metastases develop, the prognosis is poor, with very few patients living more than 1 year [7,8]. The biology of this unusual cancer is not understood and consequently, no potential targets for treatments are currently available.

Herein we report a case of metastatic malignant phyllodes tumor for which a comprehensive molecular analysis was performed by using Clinical Laboratory Improvement Amendments (CLIA)-certified labs, providing new insights into the potential opportunities for molecularly targeted therapies for this extremely rare disease.

## Methods

We reviewed the medical chart of a patient with phyllodes tumor that presented to the Department of Investigational cancer therapeutics for targeted therapy options. The patient requested a commercially available comprehensive molecular analysis by CLIA certified labs.

### Next-generation sequencing

Next-generation sequencing was performed by using the Clinical Laboratory Improvement Amendments (CLIA)-approved FoundationOne™ platform (Foundation Medicine, Cambridge, MA, USA). FoundationOne™ is a targeted assay utilizing next generation sequencing in routine cancer specimens. The test simultaneously sequences the entire coding sequence of 236 cancer-related genes (3,769 exons) plus 48 introns from 20 genes often rearranged or altered in cancer to an average depth of coverage of >250X. It detects all class of genomic alterations (including base substitutions, insertions and deletions, copy number alterations and rearrangements) using routine FFPE tissue samples that may be as small as 40 μm.

### Whole-genome array-based comparative genomic hybridization (array CGH)

Whole-genome array-based comparative genomic hybridization (array CGH) was performed by using the DNAarray™ (CombiMatrix Diagnostics, Irvine, CA). Whole-genome array-based comparative genomic hybridization (array CGH) was performed using the DNAarray™ - Breast Profile designed to detect genome-wide copy number variations. The array contains 3000+ unique large-insert clones covering coding and non-coding human genome sequences with content sourced from the UCSC hg18 human genome (NCBI build 36) and an average probe spatial resolution of ~800 Kb. DNA copy number in the patient sample was evaluated in relation to a reference diploid DNA sample, and evaluation of copy number changes was performed in all covered regions of the genome including pericentromeric, subtelomeric, and loci representative of malignant genomic changes.

### Immunohistochemical and morphoproteomics analysis

Immunohistochemical and morphoproteomics analysis were performed at Consultative proteomics^®^, The University of Texas, UT Health Medical school, Houston,TX, USA (Robert E Brown Lab); Clarient Diagnostics, Aliso Viejo, CA, USA; and Caris Life Sciences Target one, Irving, TX, USA.

### Fluorescence in situ hybridization (FISH)

FISH was performed at Clarient Diagnostics, Aliso Viejo, CA, USA. HER-2 gene amplification was assessed utilizing the PathVysion assay (Vysis Corp., Downers Grove, Illinois, USA). The identification probes for the HER-2 (SpectrumOrange) and alphasatellite DNA sequence at the centromeric region of chromosome 17 (SpectrumGreen) were hybridized according to the manufacturer’s guidelines. At least twenty non-overlapping nucleicontaining at least one orange and one green signal were enumerated. The ratio of orange signals (HER-2 gene) to green signals (chromosome 17) was calculated. A ratio greater thanor equal to 2.0 is considered as amplified based on the FDA approval in this kit. The College of American Pathologists (CAP) HER-2 consensus conference 2002 suggested that a ratio of 1.8-2.2 be considered as borderline.

## Results and discussion

### Case history

**Figure 1:**
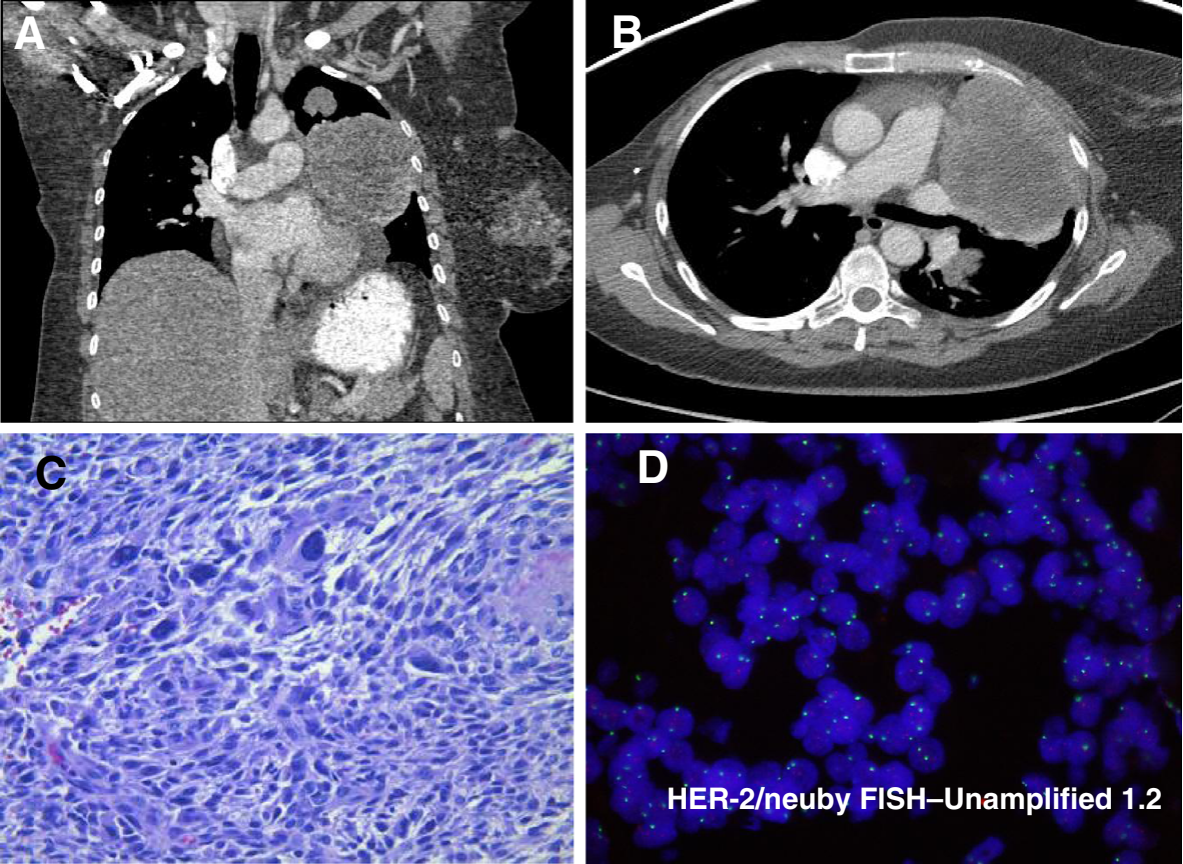
**Imaging studies, histopathology and FISH studies: UA and B:** CT of the chest showing metastatic masses in the lung. **C** Hematoxylin and Eosin stained section of the tumor from the right mastectomy shows a malignant sarcoma/malignant phyllodes tumor that has high stromal cellularity and a mitotic index of 11 per 10 high power fields. **D***HER*-*2*/*neu* by Fluorescence in Situ Hybridization (FISH) HER-2 gene amplification was assessed utilizing the PathVysion assay (Vysis Corp., Downers Grove, Illinois). The identification probes for the HER-2 (SpectrumOrange) and alpha satellite DNA sequence at the centromeric region of chromosome 17 (SpectrumGreen) were hybridized according to the manufacturer’s guidelines. At least twenty non-overlapping nuclei containing at least one orange and one green signal were enumerated. The ratio of orange signals (HER-2 gene) to green signals (chromosome 17) was calculated. A ratio greater than or equal to 2.0 is considered as amplified based on the FDA approval in this kit. The CAP HER-2 consensus conference 2002 suggested that a ratio of 1.8-2.2 be considered as borderline.

**Figure 2:**
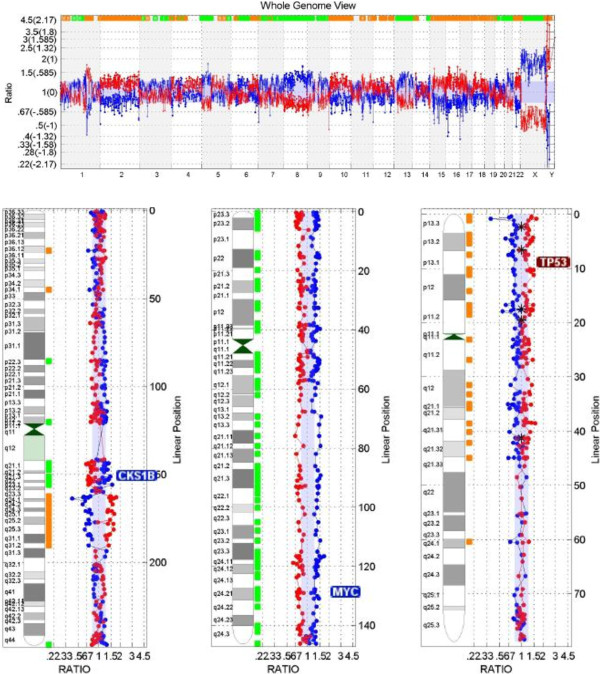
**Shows genome viewer as reported by Combimatrix positive for high level chromosomal instability (CIN).** Amplification of chromosome 1 (*CKS1B* gene), chromosome 8 (*MYC* gene), and chromosome 9 (*CDKN2A* gene) were detected. Deletions of chromosome 17 (*TP53* gene), chromosome 10 (*GATA3*), chromosome 11 (*FGF4* and *CCND1* genes) and chromosome 22 (*PDGFB* gene) were also detected.

**Figure 3:**
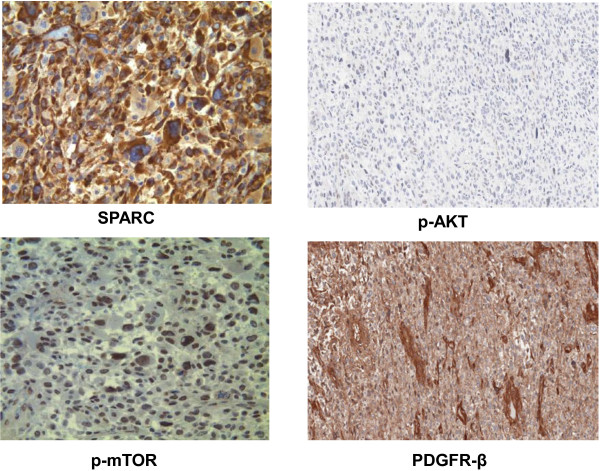
**Immunohistochemistry and morphoproteomic studies: SPARC (secreted protein acidic and rich in cysteine) is expressed at 2-3+ in the cytoplasm of the tumor cells.** The mammalian target of rapamycin (mTOR) Ser 2448 is expressed up to 2+ in the nuclei of the tumor cells. P-AKT and PDGFR β IHC staining showing an intensity of 2+.

A 55-year-old woman with a metastatic malignant phyllodes tumor presented to our clinic to discuss treatment options. Fifteen months earlier, a mass had been detected in her right breast for which a biopsy was consistent with metaplastic carcinoma/sarcoma. She underwent total mastectomy, and the diagnosis washigh-grade sarcoma arising in a phyllodes tumor with no positive lymph nodes (0/14). Immunohistochemical analysis showed that the lesion was negative for estrogen and progesterone receptor and HER-2/neu. During a follow-up examination 12 months later, computed tomography (CT) of the chest revealed five lung masses with the dominant mass measuring 6 × 4.5 × 4.5 cm within the lingula. No other sites of metastatic disease were detected. A repeated chest CT also showed multiple masses in the left lung, which is consistent with metastatic disease. The largest mass measured 10.2 × 7.6 × 8.1 cm (Figure 1A and B). Pathology review of the mastectomy product at our institution confirmed the diagnosis of a malignant phyllodes tumor characterized by stromal overgrowth of a high-grade spindle cell sarcoma with marked stromal pleomorphism, a high mitotic rate, focal cystic change, and areas of necrosis (Figure 1C). Considering the rarity of this disease and the lack of standard care options, the patient requested a commercially available comprehensive molecular analysis (Tables 1 and 2) including a whole-genome array-based comparative genomic hybridization study (array CGH) (Figures 2) and immunohistochemistry and morphoproteomics studies. (Figure 3).

**Table 1:** Summary of molecular profiling analyses from various CLIA certified methods in a patient with malignant phyllodes sarcoma

**Marker**	**Result**	**Details**
***Focused exome sequencing***		
**NRAS**	Mutated	Q61L
**TP53**	Deletion	Also proved by aCGH^1^
**RB1**	Mutated	Q504* and K740*
***Array comparative hybridization***		
**Amplifications**	Chr. 1, 8 and 9	CKS1B, MYC and CDKN2A genes
**Deletions**	Chr. 10, 11, 17 and 22	GATA3, CCND1, TP53 and PDGFB gene
***Immunohistochemistry***		
**p**-**AKT**	Positive	Intensity of 2 in 40% of cells
**PDGFRβ**	Positive	Intensity of 2 in 90% of cells
**PDGFRα**	Positive	Intensity of 2 in 80% of cells
**EGFR**	Positive	Intensity of 2 in 70% of cells
**TLE3**	Positive	Intensity of 2 in 35% of cells
**PTEN**	Present	Intensity of 2 in 90% of cells
**Kit** (**CD117**)	Negative	No staining
***Morphoproteomic immunohistochemistry***		
**mTOR**	Positive	2+ morpho test
**SPARC**	Positive	2-3+ morpho test
**ER**-**α**	Negative	Morpho test

^1^aCGH: array-based comparative genomic hybridization.

### Next-generation exome sequencing and whole-genome array-based comparative genomic hybridization

Three genes were identified with significant abnormalities: activating mutation Q61L on *NRAS*; inactivating mutations Q504* and K740* on *RB1*; and *TP53* loss. Whole-genome array-based comparative genomic hybridization (array CGH) was performed by using the CLIA-certified DNAarray™ (CombiMatrix Diagnostics, Irvine, CA). This analysis was highly positive for chromosomal instability, revealing amplifications of chromosome (chr.) 1 (*CKS1B* gene), chr. 8 (*MYC* gene), and chr.9 (*CDKN2A* gene) (Figure 2). Deletions of chr. 17 (*TP53*), chr. 10 (*GATA3*), chr. 11 (*FGF4* and *CCND1* genes), and chr. 22 (*PDGFβ*) were also detected (Figure 2). In addition, *HER*-*2*/*neu* was confirmed as negative by Fluorescence in Situ Hybridization (FISH) (Figure 1D).

### Immunohistochemical and morphoproteomics analysis

Immunohistochemical analysis for relevant markers showed a positive staining for transducing-like enhancer of split (TLE) 3; secreted protein acidic and rich in cysteine (SPARC) was expressed at 2-3+ in the cytoplasm of the tumors cells, whereas mammalian target of rapamycin (mTOR) was expressed up to 2+ in the nuclei of the tumor cells (Figure 3). Other positive relevant markers were phospho-AKT, PTEN, VEGF, PDGFR β, PDGFR α, ERCC1, and EGFR. Negative markers included ER alpha, CD117, and MGMT. All of these analyses were performed at Consultative proteomics^®^, The University of Texas, UT Health Medical School Houston,TX (Robert E Brown Lab); Clarient Diagnostics, Aliso Viejo, CA; and Caris Life Sciences Target one, Irving, TX.

## Discussion

We have reported the first complete characterization by genomic and proteomic analysis of a metastatic phyllodes tumor of the breast by commercially available profiling services (Table 2). This characterization was performed in CLIA laboratories and not in a research environment, meaning that clinical targeted therapy decisions could be made including enrollment of a patient into a clinical trial or off label use of an FDA approved agent (depending upon the molecular aberration) that could benefit this patient. With the recent increase in the number of commercially available molecular profiling services these tests are just a phone call away within the patients reach and no longer in the realm of just research publications in high impact journals. There has been an exponential increase in motivated patients with cancer who have the resources requesting these services and present to the clinic with these profiles. These present with a challenge and an opportunity for practicing oncologists. These have been useful in unraveling the biology of extremely complex and rare diseases that have no standard care therapy.

**Table 2:** **Summary of molecular aberrations in genes**, **receptors and pathways by various CLIA certified methods in a patient with phyllodes tumor**

	**RAS/RAF/MAPK**	**P13CK/AKT/mTOR**	**Cell cycle/Apoptosis/Tumor Suppressor/Other pathways**	**Chemosensitivity markers**	
***CLIA Method***,***Lab and location***				Positive markers	Negative Markers
*Foundation Next Gen Sequencing*, *Cambridge*, *Boston MA*	*NRAS*Q61L		*RB1* mutation and *TP53* loss		
*aCGHCombimatrix Array*, *Irvine*, *CA*			*CKS1B* amplification, *TP53 loss*		
***IHC*** –***Clarient***,***Aliso Viejo***, ***CA***		p-AKT		PDGFR-α and β PTEN	Her-2
*Morphoproteomics*, *UT Houston*, *TX*		p-MTOR,		SPARC	ER-alpha
*Caris Target Now*, *Irving*, *Tx*				TLE3, ERCC1	
***Potential Targeted therapy options***	**MEK inhibitors**	P13CK*/AKT*/mTOR inhibitors	**MEK inhibitors**	**Taxanes, albumin-bound paclitaxel; PDGFR inhibitors**	

Potential targeted therapy options based on best pre-clinical and/or clinical evidence are listed in the last row.

The case reported herein has several clinical features typical of metastatic malignant phyllodes tumor. Previous published reports have reported a median age at diagnosis of 50 years, the time to development of metastatic lesions between 12 and 24 months after surgery [7], and a predominance of metastasis to the lungs [7,9,10]. Moreover, this patient presented with well-characterized risk factors for the development of metastatic disease, including the presence of stromal overgrowth, mastectomy at initial surgery, larger tumor size, and high mitotic index [11].

Metastatic malignant phyllodes tumor is associated with a dismal prognosis. Mean overall survival duration in this setting is 30 months according to some series [9]. The stromal component is accepted as being responsible for the metastatic behavior, and systemic treatment often is based on guidelines for soft tissue sarcoma. Previous series showed some activity of cisplatin combined with etoposide [12] or doxorubicin [13] and of ifosfamide [14]. Nonetheless, larger series evaluating the role of adjuvant chemotherapy suggested that this subtype of breast tumor presents low sensitivity to chemotherapy [15].

Some recent studies are describing genetic changes associated with this disease. Array CGH has determined that the most frequent changes were gain of 1q and loss of 3p [16]. Interestingly, in one study, gain of 1q material was significantly associated with histologically defined stromal overgrowth and a higher likelihood of recurrence [17]. Here we described a patient presenting stromal overgrowth and a metastatic recurrence with a genetic gain in 1q associated with *CSK1B* gene amplification, Amplification and over expression of the *CSK1* gene inhibited apoptosis of cells through the *MEK*/*ERK* pathway and was associated with poor prognosis in breast cancer cells [18]. Other genetic imbalances described herein, such as gain in chr. 8 and loss in chr.10, have already been described, suggesting a high level of genomic instability in these tumors [19].

In fact, mutations in the tumor suppressor gene *TP53* appear to lead to a high level of chromosomal instability and drive oncogenesis in soft tissue sarcomas [20]. Loss of *TP53* in our patient might be associated with the higher level of chromosomal instability detected. There are no reports of p53 loss in phyllodes tumors in the Catalogue of Somatic Mutations in Cancer (COSMIC) database, although 2 of 30 patients (7%) presented with *TP53* mutations. Previous reports suggested a relationship between *TP53* expression and the malignant potential of phyllodes tumor [21,22] but the consequences of this genetic abnormality still needs to be clarified. Other genetic abnormalities in phyllodes tumor that have been described in the COSMIC database (as of February 2013) are *CDKN2A* mutation (1/25 ptes), *KIT* mutation (1/26 ptes), and *PI3KCA* mutation (1/1 pte).

Our genomic analysis reports for the first time a mutation in the *NRAS* gene in breast sarcomas. The mutation detected here (Q61L) has been shown to markedly attenuate GTP hydrolysis maintaining *NRAS* in an active GTP state [23]. Activation of this protein causes cell growth, differentiation, and survival mainly through the *RAF*/*MAPK*/*ERK* pathway [23]. Targeting this pathway with *MEK* inhibitors showed activity for patients with melanoma presenting with *NRAS* mutations [24]. Nonetheless, *NRAS* is believed to activate *PI3K* signaling in addition to the *MAPK* pathway and, indeed, we demonstrated strong expression of p-AKT and p-mTOR in this patient, suggesting concomitant activation of the *PI3K* pathway. This activation was not mediated by *PI3KCA* mutation or *PTEN* loss in this patient, indicating again a role for *NRAS*-mediated signaling. Recent evidence suggested that combining the targeting of both the *MEK*/*ERK* and *PI3K*/*mTOR* pathways might be a better strategy for the treatment of *NRAS* mutant tumors [25]. Considering both the presence of the *NRAS* mutation and *CSK1B* amplification, the use of a *MEK* inhibitor (especially with agents blocking the*PI3K* pathway) would be reasonable for this patient.

Other interesting findings in this patient were the expression of TLE3 and SPARC. The first acts downstream of beta-catenin influencing microtubule stability, and a previous study indicated that TLE3 expression was associated with improved response to taxane-based therapy in breast tumors [26]. The positivity of SPARC indicates that there may be greater delivery of albumin-bound paclitaxel to the malignant sarcoma, since SPARC is a facilitator that allows more chemotherapeutic agents to concentrate in the surrounding tumoral microenvironment [27]. Tumor responses to albumin-bound paclitaxel have already been linked to SPARC expression in some tumors [28].

The expression of estrogen receptor alpha was negative in most of the tumor, which is a well-described finding for the stromal component of phyllodes tumors [29]. Expression of both *PDGFR*α and β have already been described in phyllodes tumors and have been associated with high histologic grade and worse prognosis [30]. The therapeutic implication of this finding is not well understood, although a previous response to sunitinib, a known *PDGFR* inhibitor, in a metastatic phyllodes tumor was reported [31]. Of note, the patient who responded to sunitinib was also treated with paclitaxel, which our data showed may be active in this disease. A major limitation of this comprehensive molecular profiling is the assessment of response to molecularly targeted matched therapy. We made several treatment recommendations for this particular patient based on the discussion provided in the manuscript. These included combination of MEK inhibitors and PI3K inhibitors with or without a taxane based regimen. Unfortunately this patient came from a different country where these drugs are not available as clinical trials. In addition, due to insurance issues the patient could not be treated on our service. This is a common issue in clinic especially because insurance companies usually request a high level of evidence for allowing treatments in even rare diseases.

## Conclusion

In summary, this is the *first* report of a whole genomic profile and proteomics analysis of a metastatic phyllodes tumor of the breast. We described an *NRAS* mutation with concomitant activation of *PI3K*/*Akt*/*mTOR*, suggesting a potential role for a combination of *MEK* and *PI3K* inhibitors. We also found markers for sensitivity to taxane-based therapies, especially albumin-bound paclitaxel. Exploring the biology of rare malignancies may be a reasonable strategy for the development of targeted treatments.

## Competing interests

The authors declare no competing interests other than those mentioned in the acknowledgements section.

## Authors’ contributions

All authors contributed to writing the manuscript. DLFJ, AC and VS conceived the manuscript. AC and VS provided clinical expertise. DLFJ, AC and VS analysed the data. DLFJ, AC, VS wrote the paper. AC and VS provided sarcoma expertise. DLFJ and VS provided cellular, molecular and targeted therapy expertise. All authors read and approved the final manuscript.
